# A Case Series of Dengue Myocarditis: A Complication Observed in Dengue Patients

**DOI:** 10.7759/cureus.48285

**Published:** 2023-11-04

**Authors:** Ritika Sud, Niharika Agarwal, Varthiya Aishwarya, Anshika Aggarwal, Yogesh S, Mihit Kalawatia, Ravi Sangoi, Nida A Ahmed, Amisha Palande, Gaurav Mittal

**Affiliations:** 1 Internal Medicine, Lady Hardinge Medical College, New Delhi, IND; 2 College of Medicine, Lady Hardinge Medical College, New Delhi, IND; 3 Internal Medicine, Madras Medical College and Rajiv Gandhi Government General Hospital, Chennai, IND; 4 Internal Medicine, Rajarshee Chatrapati Shahu Maharaj Government Medical College, Kolhapur, IND; 5 Internal Medicine, Punyashlok Ahilyadevi Holkar Government Medical College and General Hospital Baramati, Baramati, IND; 6 Trauma and Orthopaedics, Barnsley Hospital National Health Service (NHS) Foundation Trust, Barnsley, GBR; 7 Physiology, Terna Medical College, Mumbai, IND; 8 Research and Development, Rotaract Club of Indian Medicos, Mumbai, IND; 9 Research, Students Network Organization, Mumbai, IND; 10 Internal Medicine, Mahatma Gandhi Institute of Medical Sciences, Wardha, IND

**Keywords:** : expanded dengue syndrome, dengue virus infection, cardiogenic shock, acute myocarditis, dengue fever

## Abstract

Dengue is a prevalent arthropod-born viral disease with a wide spectrum of clinical presentations ranging from undifferentiated fever to a more severe form of the disease, dengue hemorrhagic fever, and dengue shock syndrome. However, atypical manifestations such as hepatic, neurological, cardiac, and kidney involvement are increasingly being reported, thus the term “expanded dengue syndrome”. We report a series of cases with an atypical presentation of dengue fever marked by various cardiac manifestations, including cardiogenic shock secondary to myocardial involvement.

## Introduction

Dengue is a common arthropod-borne viral infection with a huge disease burden on Asia and the Pacific region, with more than 100 million new dengue infections every year [1-3]. Common clinical manifestations include fever, headache, arthralgia, retro-orbital pain, rash, and myalgia. A more severe clinical form is seen in dengue hemorrhagic fever (DHF) and dengue shock syndrome (DSS). DHF and DSS are characterized by increased vascular permeability, thrombocytopenia, and bleeding [1]. The unpredictable nature of the dengue virus results in the involvement of the hepatobiliary system, neurological system, and cardiovascular system. Cardiovascular involvement is usually a result of myocardial inflammation due to direct invasion by viruses and the production of inflammatory cytokines and free oxygen radicles. Myocarditis, pericardial effusion, heart block, and arrhythmias are the manifestations of cardiovascular involvement that usually follow a benign, self-limiting course but may rarely be fatal. The prevalence of dengue myocarditis is 11.2 % higher with non-severe dengue with warning signs and severe dengue (46.6%) than in non-severe dengue without warning signs (9.72%) [4,5]. Dengue, being a viral, vector-born infection, has no known treatment with high mortality and morbidity, emphasizing the role of aggressive, early detection and prevention of organ involvement [6,7]. Hence, there's an emerging role for cardiac MRI and stress echocardiography in dengue patients. We present a case series of myocarditis caused by the dengue virus.

## Case presentation

Case 1

A 27-year-old female patient was admitted during the critical phase with the usual symptoms of fever and vomiting that had resolved over four days with malaise, headache, and retro-orbital pain. There were no red-flag signs at the presentation. The examination of the admission patient was largely unremarkable. A conscious, alert patient with a blood pressure of 92/64 mmHg, pulse rate of 76 bpm, SpO2 of 99%, dextrose, or blood sugar of 126 mg. Systemic examination revealed decreased breath sounds in the right infrascapular area and epigastric tenderness with normal cardiovascular and neurological examination but subsequently had per rectal bleeding. The nonstructural protein 1 antigen (NS1Ag) was positive. The chest X-ray showed blunting of both costo-phrenic angles. The electrocardiography showed a normal sinus rhythm, and the ultrasonography revealed grade 1 fatty liver along with mild ascites. The patient was conservatively managed with intravenous fluids and random donor platelets (RDPs) following the episode of mucosal bleeding and consequently moved to the Medical Intensive Care Unit (MICU). Despite adequate hydration and cessation of bleeding, the patient had persistent tachycardia. There was a worsening of polyserositis and breathlessness. The cardiac biomarkers, N-terminal pro-b-type natriuretic peptide (NT ProBNP), and troponin I were elevated. Sinus tachycardia was documented on the electrocardiogram (ECG), and the ejection fraction (EF) was 67% on the 2D echocardiogram. Ultrasonography confirmed bilateral pleural effusion with underlying consolidation and moderate ascites. Arterial blood gas analysis (ABG) indicated respiratory alkalosis with moderate acute respiratory distress syndrome (ARDS).

**Figure 1:**
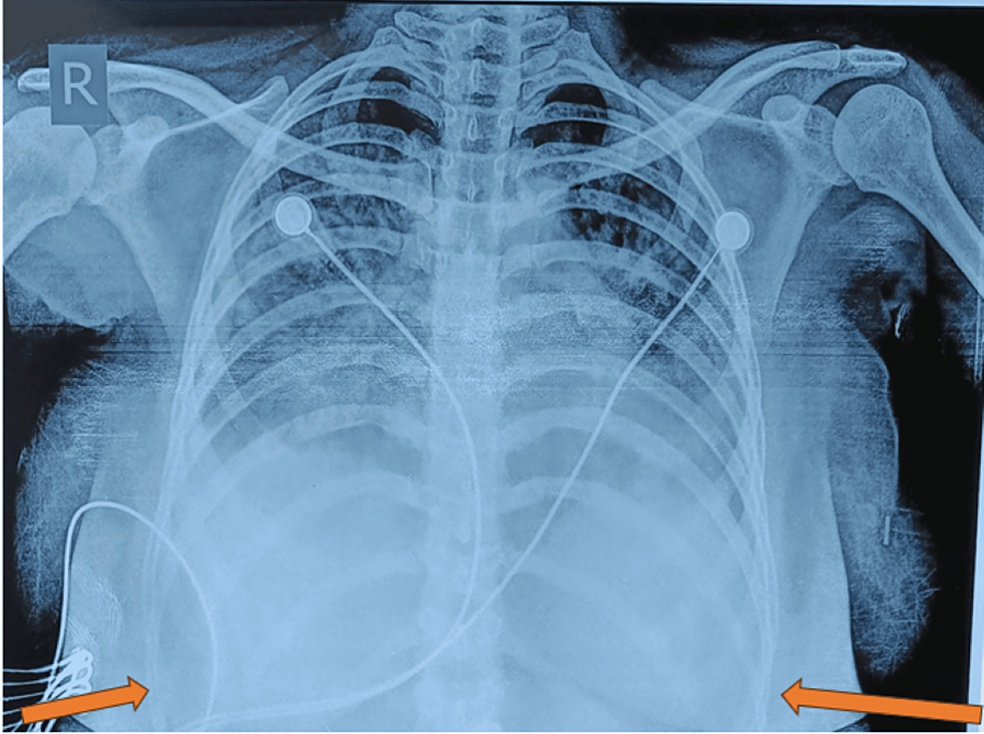
Chest X-ray of the patient showing blunting of both costophrenic angles The arrows depict the blunting of the costophrenic angle

The patient was managed for myocarditis with Enalapril, broad-spectrum antibiotics, and continuous positive airway pressure (CPAP) support. The patient received multiple blood transfusions during her hospital stay. During the course, the patient deteriorated further with poor Glasgow Coma Scale (GCS), hypoxic and hypercapnic respiratory failure, needing tracheal intubation, and mechanical ventilation. Tachypnea, tachycardia, fever, thrombocytopenia, ascites, and chest X-ray opacity resolved over the next 48 hours. She was discharged five days later. Table 1 shows the laboratory investigations of the patient at the time of admission and on day four of admission. The chest X-ray of the patient shows blunting of both costophrenic angles, which can be seen in Figure 1. 

**Table 1:** Laboratory investigations of patient at the time of admission and at day four of admission TLC: Total leukocyte count, CK-MB: Creatine kinase-myoglobin binding, SGOT: Glutamic-oxaloacetic transaminase, SGPT: Glutamic-pyruvic transaminase, NT-proBNP: N-terminal pro b-type natriuretic peptide

Laboratory test	Hospital admission	Day 4	Normal range
Hemoglobin[g/dl]	12.1	9.1	12-16g/dL
Hematocrit %	35.2	26.5	36-44%
TLC	2400	22000	4000-11000/microliters
Platelets	25000	480000	150000-450000/microliters
Creatinine	0.8	0.6	0.8-1.3 mg/dL
Troponin I		0.3	0-0.04ng/mL
CK-MB[U/L]	29	53	5-25IU/L
Lactate [mmol/l]	1.5	2.1	<2mmol/L
C-reactive protein[mg/dl]	29.1	66.2	<0.3mg/dL
SGOT/SGPT	289/68	121/96	5-40/7-56IU/L
Serum ferritin		846	12-300ng/mL
Serum fibrinogen		431	200-400mg/dL
NT- proBNP		8008	<125pg/mL
Dengue NS1	Positive		-
Dengue IgM		Positive	-

Case 2

A 44-year-old female with a known case of hypothyroidism was admitted with an episode of hematemesis, which was preceded by a high-grade fever and headache for the last six days. At admission, the patient was conscious, dehydrated, and afebrile, with a BP of 112/60 mmHg, a pulse rate of 100 bpm, and a SpO2 of 98%. On systemic examination, per abdomen - epigastric tenderness and tachycardia with no respiratory or neurological involvement. Ultrasonography showed grade 1 fatty liver with pseudoedema of the gall bladder and mild ascites. The chest X-ray was normal, and ECG was suggestive of sinus tachycardia. Hypovolemic shock resolved after fluid restoration with 0.9% normal saline. A provisional diagnosis of dengue fever with hypovolemic shock with primary hypothyroidism was managed conservatively. Dengue IgM was positive, and myocarditis was suspected due to persistent tachycardia with raised cardiac biomarkers. The patient was managed symptomatically, improved without any complications, and was discharged after six days of hospital stay. Table 2 shows the laboratory investigations of the patient at the time of admission and on day three of admission. 

**Table 2:** Laboratory investigations of the patient at the time of admission and at day three of admission TLC: Total leukocyte count, CK-MB: Creatine kinase-myoglobin binding, SGOT: Glutamic-oxaloacetic transaminase, SGPT: Glutamic-pyruvic transaminase, NT-proBNP: N-terminal pro b-type natriuretic peptide

Laboratory tests	Hospital admission	Day 3	Normal range
Hemoglobin[g/dl]	12.4	10	12-16g/dL
Hematocrit %	35.7	27.9	36-44%
TLC	1200	6400	4000-11000/microliters
Platelets	8000	37000	150000-450000/microliters
Creatinine	0.5	0.6	0.8-1.3 mg/dL
CK-MB[U/L]		86	5-25IU/L
Lactate [mmol/l]		1.18	<2mmol/L
C-reactive protein[mg/dl]	23	25	<0.3mg/dL
SGOT/SGPT	424/107	1981/606	5-40/7-56IU/L
Serum ferritin		846	12-300ng/mL
Serum fibrinogen		431	200-400mg/dL
NT- proBNP[pg/dl]		239	<125pg/mL
Dengue NS1	Negative		-
Dengue IgM		Positive; raised titers	-

Case 3

**Figure 2:**
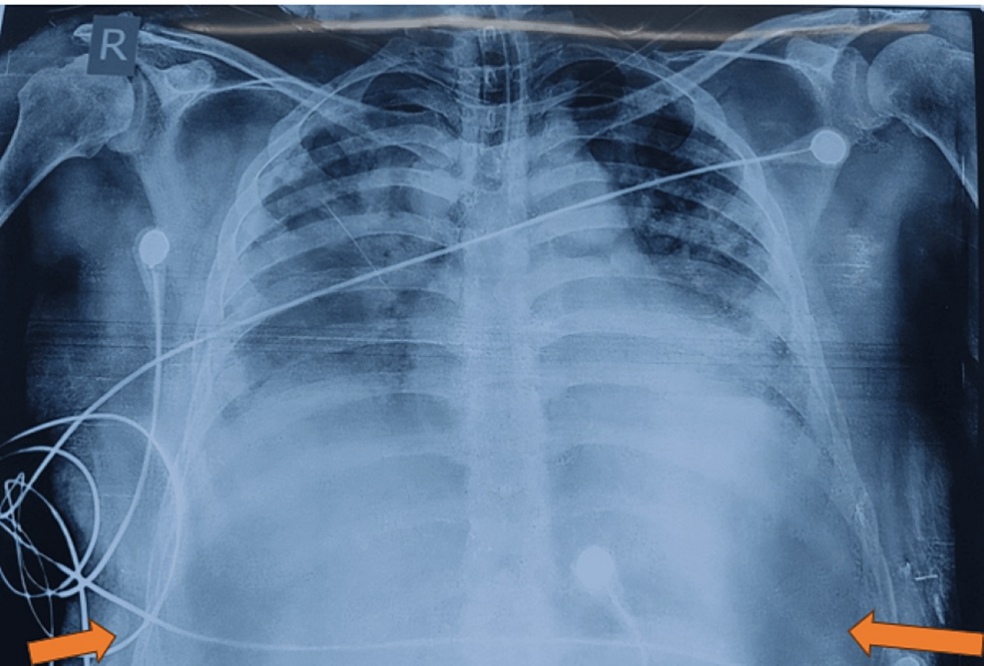
Chest X-ray of the patient at the time of admission The arrows depict the blunting of the costophrenic angle.

A 28-year-old male presented with complaints of fever for five days with headaches and malaise. Vomiting, pain in the abdomen, hematochezia, and dizziness for the last two days. He was afebrile for the last 24 hours. The patient was on medication for rheumatoid arthritis (ADALIMUMAB every 15 days). On clinical examination at admission, the patient was conscious and well-oriented, with a BP of 106/86 mmHg, a pulse rate of 88 bpm, a SpO2 of 98% on room air, and a dextrose or blood sugar of 136 mg/dl. No significant systemic abnormality was present. Figure 2 shows laboratory investigations of the patient at the time of admission and on day three of admission. On evaluation, prothrombin time was 18.8 seconds, with an international normalized ratio of 1.66. The ECG showed normal sinus rhythm. On further investigation, the Widal test was negative, and the NS1 antigen was positive. A diagnosis of Dengue Hemorrhagic Fever was made and managed conservatively. The next day patient went into shock [BP - 90/60mmHg, PR - 160 bpm] with peripheral cyanosis, cold and clammy skin, capillary refilling time >3 secs with diffuse blanchable rash. ECG showed sinus tachycardia. Fluid restoration, infusion of sodium bicarbonate, electrolyte correction, and vasoactive drugs were promptly initiated. Troponin I was positive, cardiac biomarkers were raised with coagulopathy and deranged hepatic and renal function test, and ABG was suggestive of high anion gap metabolic acidosis (HAGMA). A diagnosis of dengue hemorrhagic fever with viral myocarditis, cardiogenic shock with multiple organ dysfunction, and severe acidosis was made. Blood transfusion, fresh frozen plasma, and random donor platelets transfused. Due to respiratory distress, the patient was electively intubated, and mechanical ventilation was initiated. He deteriorated further and went into bradycardia, followed by sudden cardiac arrest. Cardiopulmonary resuscitation was performed without success. Table 3 depicts the chest X-ray of the patient at the time of admission. 

**Table 3:** Laboratory investigations of the patient at the time of admission TLC: Total leukocyte count, CK-MB: Creatine kinase-myoglobin binding, SGOT: Glutamic-oxaloacetic transaminase, SGPT: Glutamic-pyruvic transaminase, NT-proBNP: N-terminal pro b-type natriuretic peptide

Laboratory test	Hospital admission	Normal range
Hemoglobin[g/dl]	13.6	14-18g/dL
Hematocrit %	38.1	41-50%
TLC	5140	4000-11000/microliters
Platelets	95000	150000-450000/microliters
Creatinine	1.4	0.8-1.3 mg/dL
Troponin I	0.26	0-0.04ng/mL
Lactate [mmol/l]	10.04	<2mmol/L
C-reactive protein[mg/dl]	32.2	<0.3mg/dL
SGOT/SGPT	4100/4500	5-40/7-56IU/L
Serum ferritin	>15000	12-300ng/mL
Serum fibrinogen	431	200-400mg/dL
NT- proBNP	1106	<125pg/mL
Dengue NS1	Positive	-
Dengue IgM	Positive; raised titers	-

## Discussion

Dengue is a global problem affecting more than 40% of the world's population living in endemic areas. According to a WHO report, 2022 saw a two-fold increase in dengue cases worldwide. The National Center for Vector-Borne Disease Control in India reported 2,33,251 cases, with 303 deaths in 2022. The prevalence of myocarditis in hospital dengue patients is 4.2%, and all patients had raised cardiac troponin I [cTn-I]. 59.5% had at least one change on the ECG, and 24% had a reduced ejection fraction (EF <55%). It was observed that patients with abnormal ECG had a prolonged hospital stay (three days) and that raised cTn I was significantly associated with in-hospital mortality [8,9]. In a study in Sri Lanka, 25% of patients had one or more elevated cardiac biomarkers of myocardial injury, like myoglobin, CK-MB, troponin, and NT pro-BNP. In a case report, a 37-year-old woman with dengue developed fulminant and fatal cardiogenic shock with evidence of raised troponin I, CK-MB, and myocardial biopsy [7]. In a study of 81 patients, 18 had cardiac involvement, three had left ventricular systolic dysfunction, three had transient diastolic dysfunction, six had increased levels of at least one cardiac biomarker (troponin, CKMB), and six had pericardial involvement. It was observed that cardiac involvement is more common in DHF than in dengue fever. We report a series of cases with cardiac involvement and evidence of raised cardiac biomarkers and ECG changes. A 28-year-old female with DHF with myocarditis and cardiac failure required respiratory support and a prolonged ICU stay before eventually recovering and being discharged [8]. Another case of a 44-year-old female with DHF with myocarditis recovered with supportive care. A 28-year-old male DHF with myocarditis progressed rapidly to cardiogenic shock and sudden cardiac arrest. The clinical presentation of dengue patients may vary from typical symptoms like thrombocytopenia, fever, weakness, fatigue, and vomiting to severe atypical symptoms like myocarditis. The above-discussed patients, despite having no history of cardiac problems, showed sinus tachycardia on a 12-lead ECG. The cardiac markers were also elevated (troponins). As per European Society of Cardiology (ESC) guidelines, a diagnosis of myocarditis was made [9]. This complication of dengue fever can further lead to cardiac arrest and shock, and it is fatal. Further findings, like structural abnormalities in cardiac imaging and cardiovascular magnetic resonance (CMR), can be done to confirm the diagnosis. Moreover, a common finding found in all three cases was elevated ferritin levels after blood transfusion, which can lead to inflammation and cardiac dysfunction [10]. Due to the lack of a specific treatment for the dengue virus, these complications need to be explored so that advancements can occur in the existing treatment and, hence, we can decrease the mortality rate [11]. Considering the major limitation of the existing literature, i.e., the absence of proper early diagnosis of dengue myocarditis in young patients, this study provides significant evidence to diagnose early dengue myocarditis in the young adult population [12,13].

## Conclusions

The escalating prevalence of dengue cases underscores the critical need for a heightened index of suspicion when diagnosing complications, especially myocarditis. Given its often asymptomatic presentation and dire prognosis, it is imperative to exercise vigilance in clinical settings. Early detection and intervention are pivotal in averting further detrimental consequences.

Myocarditis, a rare but severe manifestation of dengue fever, necessitates a swift and accurate diagnosis. As symptoms can be subtle or absent, physicians must maintain a high level of suspicion, particularly in regions endemic to dengue. The silent progression of myocarditis can swiftly lead to cardiac complications, emphasizing the urgency of early detection. Patients with dengue, even in the absence of overt cardiac symptoms, should be closely monitored, and cardiac evaluation should be a routine part of the diagnostic protocol.
